# Furunculas

**Published:** 1854-06

**Authors:** 


### *Furunculus.*

In the furunculoid epidemic which has lately prevailed so extensively throughout the world, various means are proposed by different medical men; nitro-muriatic acid, alterative doses of mercury, iron, chlorate of potash, yeast and quinine, are among the chief measures recommended. Upon the hands this affection usually assumes the form of paronychia, which we have frequently found to yield to an abortive treatment, consistng in the application of half an ounce of strong mercurial ointment and two drachms of extract of belladonna. —*Virginia Med. & Surg. Jour.*

